# Imaging Findings, Fluoroscopic Time, and Results of the Lumbosacral Selective Nerve Root Block: Focus on the L5 Nerve Root Block

**DOI:** 10.7759/cureus.43872

**Published:** 2023-08-21

**Authors:** Hisashi Serikyaku, Shoichiro Higa, Tetsuya Yara

**Affiliations:** 1 Department of Orthopaedics, Naha City Hospital, Naha, JPN

**Keywords:** radiation exposure, lumbar degenerative disease, radiating pain, fluoroscopic time, selective nerve root block

## Abstract

Introduction

Selective nerve root block (SNRB) is a valuable diagnostic and therapeutic tool. In some cases, intra-nerve root puncture is difficult and time-consuming, and radiation exposure time for the surgeon may be prolonged. The aim of this study is to examine the contrast findings, fluoroscopic time, and outcomes of SNRB.

Methods

A total of 139 cases of SNRB were included in the study. We investigated radiating pain presence, duration of fluoroscopic time, contrast types for nerve roots, and SNRB outcomes. Contrast patterns of nerve roots were categorized into three types, which were: type 1: the presence of contrast along the nerve roots; type 2: the presence of contrast within the intravertebral foramen but not in the nerve root; and type 3: the absence of both nerve root and intravertebral foramen contrast.

Results

The mean fluoroscopic time was 12.8 ± 15.3 seconds for type 1, 11.1 ± 8.9 seconds for type 2, and 23.6 ± 18.8 seconds for type 3. Statistically significant differences were found between the three groups (p = 0.007), and subsequent multiple comparisons showed significant differences between type 1 and type 2 (p = 0.010) and between type 2 and type 3 (p = 0.015). The visual analog scale (VAS) score before and 30 minutes after SNRB demonstrated a significant improvement in all patients. The mean change in VAS before and after nerve root block was 49.6 ± 21.7 mm for type 1 cases, 49.8 ± 25.2 mm for type 2 cases, and 37.8 ± 23.6 mm for type 3 cases, with no statistically significant difference between the three groups (p = 0.090). The proportion of patients with subjective symptomatic improvement before and after SNRB was 91.3% in type 1 cases, 88.5% in type 2 cases, and 85.7% in type 3 cases, with no statistically significant difference between the three groups (p = 0.641).

Conclusions

The above findings indicate that type 3 is beneficial for both diagnostic and therapeutic purposes.

## Introduction

Selective nerve root block (SNRB) is a valuable diagnostic and therapeutic tool for managing nerve root symptoms associated with lumbar degenerative diseases. Further, SNRB is a treatment option that lies between conservative treatment with analgesics and surgical treatment [1-3].

Obtaining clear nerve root imaging and radiating pain is generally considered desirable when performing SNRB. Nonetheless, certain factors, such as bone spurs, scoliosis, and spinal rotation, may render it challenging to achieve clear imaging and pain radiation in some cases. Prolonged fluoroscopic procedures raise concerns about X-ray exposure.

If the nerve roots are difficult to reach and the fluoroscopy time is prolonged, the procedure may have to be terminated by administering a blocking agent around the nerve root without injecting it into the nerve root.

There is a report of a 1.9-fold increased prevalence of cancer and a 2.9-fold increased prevalence of breast cancer in female orthopaedic surgeons compared with U.S. women of similar age and race [4]. Furthermore, it has been reported that the orthopaedic surgeon has a 5.37-fold higher incidence of cancer compared to other unexposed medical workers [5]. Spinal surgeons are reported to have 3.2 times more skin disorders compared with other orthopaedic surgeons [6], so reducing radiation exposure is an urgent issue for orthopaedic surgeons, especially those involved in spine surgery.

The aim of this study is to examine the contrast findings, fluoroscopic time, and outcomes of SNRB to determine the extent to which intra-nerve root puncture should be pursued and whether a reduction in radiation exposure can be obtained.

## Materials and methods

The subject was a case of SNRB of the fifth lumbar spinal nerve root performed in Naha City Hospital, Naha, Japan, by the same operator (author) in the period from October 2017 to July 2022, in a case where the fifth lumbar spinal nerve root was caused by a lumbar degenerative disease. Cases of a second or subsequent block and cases without MRI imaging were excluded. There were 139 cases of 78 male and 61 female patients, and the mean age at the time of the SNRB was 62.9 ± 17.2 (11-92) years. The breakdown of the diseases was as follows: 69 cases of lumbar degenerative foraminal stenosis, 28 cases of lumbar spinal stenosis, 26 cases of lumbar disc herniation, five cases of foraminal stenosis accompanying isthmic spondylolysis/spondylolisthesis, three cases of postoperative recurrent lumbar spinal stenosis, and eight cases of recurrent lumbar disc herniation. Approval to conduct the study was taken from the Institutional Review Board (IRB) of Naha City Hospital (approval number: 2023a19)

We conducted an investigation to evaluate the presence of radiation pain in the same area as the symptoms, fluoroscopic time, contrast type for nerve roots, and the outcomes of SNRB. The changes in the visual analog scale (VAS) and subjective symptoms before and 30 minutes after the block were also assessed. We used a standard 100-mm horizontal bar VAS scale on which the left border (0 mm) was denoted as "no pain" and the right (100 mm) as "worst pain imaginable". The nerve root puncture was attempted using a 25-gauge spinal needle under fluoroscopy guidance. The blocking agent used in this study consists of a combination of 1 ml of lidocaine hydrochloride (1.0%) and 1 ml of dexamethasone phosphate sodium (1.65 mg).

**Figure 1:**
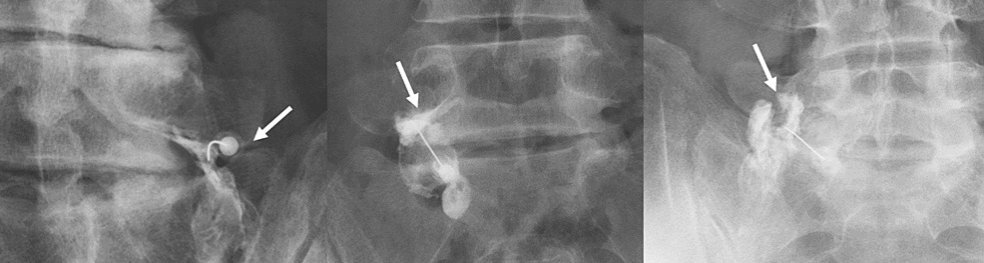
The contrast patterns of the SNRB SNRB: selective nerve root block Type 1 is characterised by the presence of contrast along the nerve root (left image; arrow); type 2 is characterised by the absence of nerve root contrast but the presence of contrast within the intravertebral foramen (middle image; arrow); and type 3 is characterised by the absence of both nerve root contrast and intravertebral foramen contrast (right image; arrow).

We categorised contrast patterns of the nerve roots into three distinct types, which were: type 1: characterised by the presence of contrast along the nerve roots; type 2: characterised by the absence of nerve root contrast but the presence of contrast within the intravertebral foramen; and type 3: characterised by the absence of both nerve root contrast and intravertebral foramen contrast (Figure 1).

The authors have implemented strategies to mitigate X-ray exposure to the operator, including the use of radiation-protective clothing, eyeglasses, and thyroid collars. Additionally, the surgeon performs a fluoroscopy and takes one shot.

Statistical analyses

All statistical analyses were performed using IBM SPSS Statistics software version 22.0 (IBM Corp., Armonk, NY, USA). A paired Student’s t-test was used for the comparison of continuous variables, and an X^2^ or Fisher’s exact test was used for the comparison of categorical variables. An analysis of variance (ANOVA) tool was used for comparisons of continuous variables among the three groups. A p-value < 0.05 was considered statistically significant in all analyses.

## Results

Among the observed cases, type 1 accounted for 92 cases (66.2%), type 2 for 26 cases (18.7%), and type 3 for 21 cases (15.1%). The mean age of cases was 63.6 ± 17.1 years for type 1, 58.8 ± 18.7 years for type 2, and 64.7 ± 15.8 years for type 3, with no significant differences between the three groups (p = 0.396). The sex distribution among the three groups was as follows: in type 1 cases, there were 51 males and 41 females; in type 2 cases, 16 males and 10 females; and in type 3 cases, 11 males and 10 females, with no significant differences in the sex ratios between the three groups (p = 0.793). The breakdown of diseases in the three groups is shown in Table 1.

**Table 1:** Demographic data for each group

	Type 1 (n=92)	Type 2 (n=26)	Type 3 (n=21)	P-value
Age (years)	63.6±17.1	58.8±18.7	64.7±15.8	0.396
Sex (n)	51/41	16/10	11/10	0.793
Diseases				N.S
Lumbar foraminal stenosis (n)	44	9	15	
Lumbar spinal stenosis (n)	21	6	2	
Lumbar disc herniation (n)	17	7	2	
Lumbar isthmic spondylosis/spondylolisthesis (n)	3	0	2	
Lumbar spinal restenosis (n)	1	2	0	
Lumbar disc reherniation (n)	6	2	0	

Overall, the incidence of radiating pain at the same site as the symptoms was 61.9%. Type 1 was 56.5%, type 2 was 65.4%, and type 3 was 47.6%, with no statistically significant differences between the three groups (Table 2).

**Table 2:** Measurement results for each group VAS: visual analog scale

	All (n=139)	Type 1 (n=92)	Type 2 (n=26)	Type 3 (n=21)	P-value
Radiating pain (%)	61.9%	56.5%	65.4%	47.6%	0.377
Mean fluoroscopic time (sec)	14.1±15.4	12.8±15.3	11.1±8.9	23.6±18.8	0.007
Pre-block VAS (mm)	68.6±15.1	69.2±15.1	71.5±12.7	62.2±16.3	0.085
Post-block VAS (mm)	20.8±19.2	19.7±18.4	21.6±20.4	24.4±21.1	0.575
Mean change in VAS (mm)	47.8±22.9	49.6±21.7	49.8±25.2	37.8±23.6	0.090
Improvement (%)	89.9%	91.3%	88.5%	85.7%	0.641

The mean duration of fluoroscopic time was 14.1±15.4 seconds overall. Type 1 cases were 12.8 ± 15.3 seconds; type 2 cases were 11.1 ± 8.9 seconds; and type 3 cases were 23.6 ± 18.8 seconds. Statistically significant differences were found between the three groups (p = 0.007) (Table 2), and subsequent multiple comparisons showed significant differences between type 1 cases and type 2 cases (p = 0.010) and between type 2 and type 3 cases (p = 0.015) (Table 3).

**Table 3:** Multiple comparisons for mean fluoroscopic time

	Type 1 vs. Type 2	Type 1 vs. Type 3	Type 2 vs. Type 3
P-value	1.000	0.010	0.015

The VAS scores before and 30 minutes after SNRB demonstrated a significant improvement in all patients, with scores decreasing from a mean of 68.6 ± 15.1 mm to 20.8 ± 19.2 mm (p<0.001). Specifically, for type 1 cases, the VAS scores improved from 69.2 ± 15.1 mm to 19.7 ± 18.4 mm (p<0.001), for type 2 cases from 71.5 ± 12.7 mm to 21.6 ± 20.4 mm (p<0.001), and for type 3 cases from 62.2 ± 16.3 mm to 24.4 ± 21.1 mm (p<0.001).

In all three types, the improvements were statistically significant, indicating a notable reduction in pain intensity after the nerve root block procedure. The mean change in VAS before and after nerve root block was 47.8 ± 22.9 mm overall, 49.6 ± 21.7 mm for type 1 cases, 49.8 ± 25.2 mm for type 2 cases, and 37.8 ± 23.6 mm for type 3 cases, with no statistically significant difference between the three groups (p = 0.090). The proportion of patients with subjective symptomatic improvement before and after SNRB was 89.9% in all patients, 91.3% in type 1 cases, 88.5% in type 2 cases, and 85.7% in type 3 cases, with no statistically significant difference between the three groups (p = 0.641).

## Discussion

Selective nerve root block is a therapeutic method performed by injecting a needle tip into the spinal nerve exiting the spinal canal through the intervertebral foramen, followed by checking for radiating pain to the dermatome of the area innervated by the nerve, and then injecting local anaesthetics with or without steroids to alleviate the symptoms. At the same time, it has the diagnostic value of identifying the responsible nerve root [1-3].

Selective nerve root block is frequently used, especially in the lumbosacral region, and the main target diseases are radiculopathy associated with lumbar spinal canal stenosis, lumbar disc herniation, foraminal stenosis with isthmic or degenerative spondylolisthesis, and vertebral fracture [1,2,3].

Among the aforementioned disorders, there are those characterised by spinal rotation and osteophytic hypertrophy, which frequently result in unsuccessful needle penetration into the affected nerve. However, insistence on direct needle tip penetration into the affected nerve can sometimes prolong fluoroscopic time. In recent years, medical X-ray exposure has become an issue, and the limits of radiation exposure to medical personnel are clearly defined as occupational exposure [7]. Excessive exposure to X-rays may cause skin disorders, cataracts, and cancer [8, 9], underscoring the significance of minimising radiation dosage.

Today, patients are benefiting from minimally invasive spine surgery, facilitated by techniques such as percutaneous pedicle screw (PPS) fixation and lateral access lateral lumbar interbody fusion (LLIF). As minimally invasive surgery (MIS) advances, the prolonged duration of exposure to radiation is increasing. The mean fluoroscopic duration per single-level LLIF was reported to be 88.3~201.8 seconds [10,11,12]. The mean radiation exposure time of PPS per segment was reported to be significantly longer than that of conventional open pedicle screw (PS) fixation [13]. Rampersaud reported a mean fluoroscopic time of 9.3 seconds per screw insertion for the conventional open PS [14], whereas Mroz reported a mean of 29 seconds for the PPS [15].

The authors have implemented strategies to mitigate X-ray exposure to the operator, including the utilisation of radiation-protective clothing, eyeglasses, and thyroid collars. Additionally, the surgeon performs a fluoroscopy and takes one shot.

In this study, we evaluated the reproducibility of symptoms and the effectiveness of a nerve root-blocking agent administered to patients unable to undergo direct nerve root puncture due to factors such as lumbar rotation, scoliosis, or osteophytes (type 3). There were no significant differences between the three groups in terms of symptom reproducibility, and there were no significant differences in the degree of change evaluated by VAS between the three groups. Furthermore, there was no statistically significant difference observed among the three groups regarding the proportion of patients reporting subjective symptom improvement 30 minutes after the L5 nerve root block. The above results suggest that even type 3 may have diagnostic and therapeutic value.

A plausible mechanism for the radiating pain observed in type 3 cases, localised in the same area as the symptoms, could be attributed to the involvement of the medial branch of the posterior branch of the fifth lumbar nerve [16].

Periradicular infiltration of blockade into the nerve root, which corresponds to type 2 cases in this study, is not a method of injecting blocking agents directly into the nerve, but it has been shown to be effective [17]. The amelioration of pain in type 3 cases could be attributed to the spreading and penetration of the blocking agent reaching the nerve root. In a study on the diffusion of blocking agents (0.5 ml) in the block of the medial branch of the posterior branch of the lumbar spinal nerve, Dreyfuss reported that diffusion into the spinal canal and intervertebral foramen was observed in six out of 30 cases in the block of the medial branch of the posterior branch of the L5 nerve root [18].

Limitations

One limitation of our study is that we solely measured the fluoroscopic duration and did not investigate the radiation exposure dose to the surgeon. Future studies are warranted to comprehensively evaluate both fluoroscopic duration and radiation exposure to the surgeon during SNRB.

## Conclusions

The above findings indicate that type 3 is beneficial for both diagnostic and therapeutic purposes. Consequently, in situations where nerve root puncture during SNRB proves to be time-consuming, it might be recommended to consider discontinuing the procedure in type 3 cases to minimise radiation exposure. However, it is essential to make every effort to ensure an accurate puncture within the nerve root to achieve optimal outcomes for the patient.
